# An integrative model of the intrinsic hippocampal theta rhythm

**DOI:** 10.1371/journal.pone.0182648

**Published:** 2017-08-07

**Authors:** Ali Hummos, Satish S. Nair

**Affiliations:** 1 Department of Health Informatics, University of Missouri, Columbia, Missouri, United States of America; 2 Department of Psychiatry, University of Missouri, Columbia, Missouri, United States of America; 3 Department of Electrical & Computer Engineering, University of Missouri, Columbia, Missouri, United States of America; SUNY Downstate MC, UNITED STATES

## Abstract

Hippocampal theta oscillations (4–12 Hz) are consistently recorded during memory tasks and spatial navigation. Despite several known circuits and structures that generate hippocampal theta locally *in vitro*, none of them were found to be critical *in vivo*, and the hippocampal theta rhythm is severely attenuated by disruption of external input from medial septum or entorhinal cortex. We investigated these discrepancies that question the sufficiency and robustness of hippocampal theta generation using a biophysical spiking network model of the CA3 region of the hippocampus that included an interconnected network of pyramidal cells, inhibitory basket cells (BC) and oriens-lacunosum moleculare (OLM) cells. The model was developed by matching biological data characterizing neuronal firing patterns, synaptic dynamics, short-term synaptic plasticity, neuromodulatory inputs, and the three-dimensional organization of the hippocampus. The model generated theta power robustly through five cooperating generators: spiking oscillations of pyramidal cells, recurrent connections between them, slow-firing interneurons and pyramidal cells subnetwork, the fast-spiking interneurons and pyramidal cells subnetwork, and non-rhythmic structured external input from entorhinal cortex to CA3. We used the modeling framework to quantify the relative contributions of each of these generators to theta power, across different cholinergic states. The largest contribution to theta power was that of the divergent input from the entorhinal cortex to CA3, despite being constrained to random Poisson activity. We found that the low cholinergic states engaged the recurrent connections in generating theta activity, whereas high cholinergic states utilized the OLM-pyramidal subnetwork. These findings revealed that theta might be generated differently across cholinergic states, and demonstrated a direct link between specific theta generators and neuromodulatory states.

## Introduction

Slow oscillations at theta frequencies (4–12 Hz) are consistently recorded in the hippocampus during working memory tasks, spatial navigation, and storage of episodic memory [for review, see 1,2]. The hippocampus is capable of generating its own theta rhythm when isolated *in vitro* [3,4], and several structures and circuits have been identified as potential intrinsic generators of hippocampal theta [for review, see 1,2]. However, experiments aimed at confirming the role of these structures individually have invariably revealed conditions where the structures made no contribution to hippocampal theta. For instance, the slow firing oriens-lacunosum moleculare (OLM) cells, which lock closely to theta rhythm *in vivo* [5], were proposed as generators of the rhythm, using computational models [3,6]. However, later experiments showed that OLM cells possess modest resonance at theta frequencies [7], and their silencing in vivo did not diminish theta activity [8]. As a second example, computational models have suggested a contribution to hippocampal theta from intrinsic membrane conductances such as the spike-frequency adaptation currents [9–13], or the h-current [3,6,14–17]. Spike-frequency adaptation currents remain difficult to investigate experimentally, while a genetic knockout of the h-current (HCN1 channels) did not disrupt theta [18,19]. A third theta generator implicated by models is the recurrent excitatory connections between pyramidal cells [9,10,20–23]; experiments again revealed persistent theta oscillations despite disruption of this excitatory glutamatergic transmission in CA1 [24,25]. These observations might indicate a cooperative interaction between the proposed generators of theta, but previous modelling studies have typically focused on a limited set of these generators, and several questions remained unanswered, such as the extent to which each generator contributes to theta power, and whether their relative contributions change in different behavioral or neuromodulatory states.

In addition, despite the presence of these intrinsic hippocampal generators, external input plays a major role and hippocampal theta is severely attenuated *in vivo* by disruption of the input from the medial septum [26–30] and from the entorhinal cortex (EC) [31]. The contribution of input from medial septum and EC to hippocampal theta is assumed to be a consequence, solely, of the rhythmic nature of these external inputs, or the specific delays in the feedback loops formed between these external inputs and the hippocampus [32], but the hippocampus also receives input with less prominent rhythmic modulation, (for e.g. from the lateral EC, compared to the medial EC [33]). Non-rhythmic random spiking arriving through divergent afferent projections to an area has been implicated in oscillations in models [34–36] and in experiments involving the olfactory cortex [37], but has not been investigated for the hippocampus. Modeling allowed us to dissociate and examine how the non-rhythmic component of input from the medial septum and EC might also contribute to hippocampal theta.

We used our previously developed biophysical computational model of the hippocampus [38] that included principal cells and two types of interneurons, to shed light on the cooperative interactions amongst the various intrinsic theta generators, and to examine their relative contributions to the power of hippocampal theta, across neuromodulatory states. The model included neuromodulatory inputs, spatially realistic connectivity, and short-term synaptic plasticity, all constrained by prior experimental observations. To isolate the role of the non-rhythmic component of medial septal and EC inputs in generating theta, we used an input layer of neurons (referred to henceforth as ‘EC’) excited by random noise constrained by realistic hippocampal unit firing rates. We demonstrated five generators of theta power in our model, as previously reported in the literature, and found that these generators operated simultaneously and cooperatively and no one generator was critical to the theta rhythm. We then quantified their relative contribution to theta power using tractable analysis that maintains relevance to experiments. The non-rhythmic external input had the highest contribution to theta power, which is consistent with the significant drop in theta power following removal of medial septum [29] or EC inputs [31] to the hippocampus *in vivo*. Contributions from two theta generators were dependent on cholinergic state. Low cholinergic states engaged the recurrent connections amongst pyramidal cells for theta generation, while high cholinergic states utilized the OLM-pyramidal cells subnetwork, indicating that the low and the high cholinergic states had distinct mechanisms for theta generation, with specific cholinergic effects fostering the engagement of certain theta generators.

## Results

**Fig 1:**
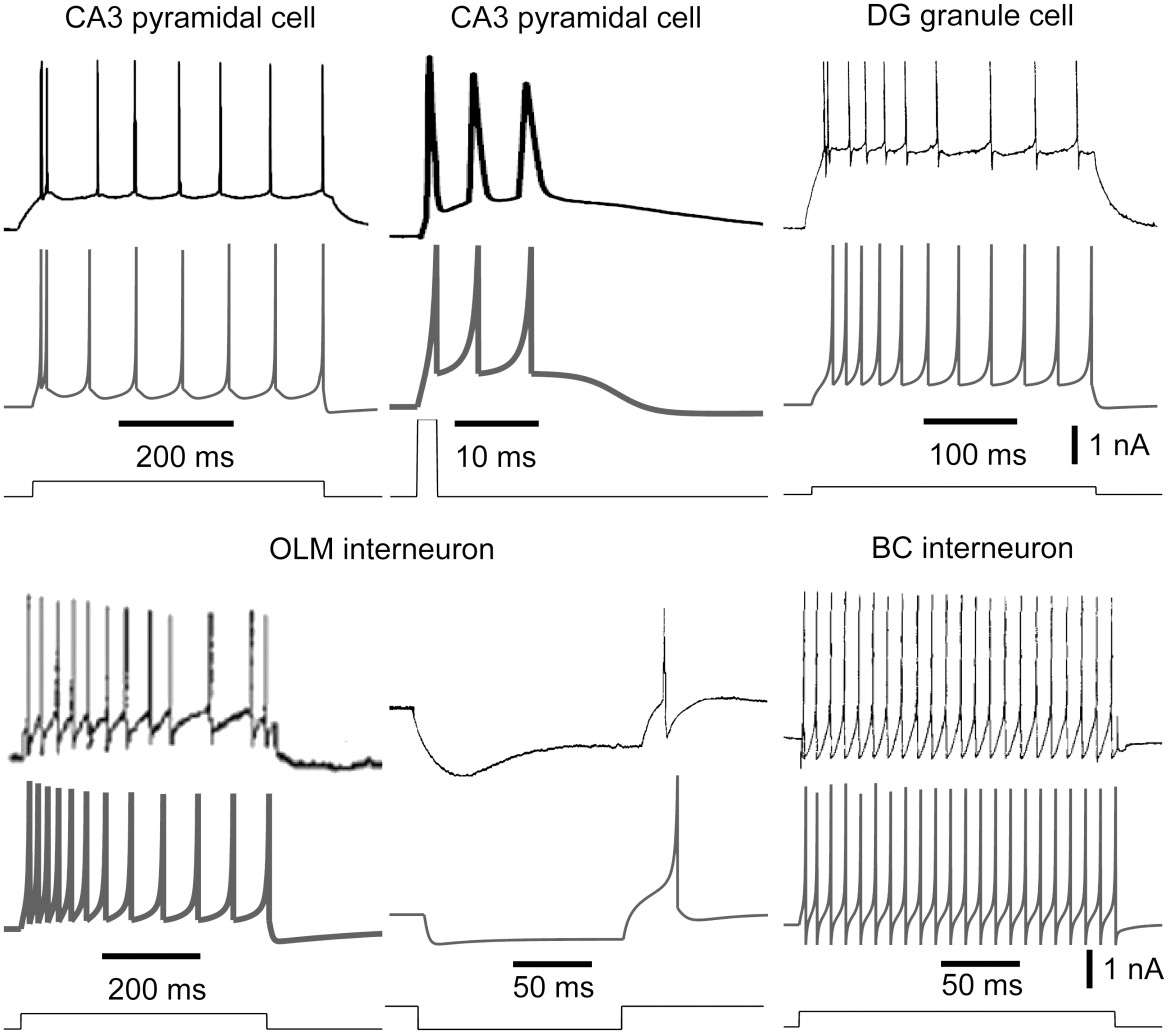
In vitro current injection recordings of cell types and of the corresponding model cells. For each cell type we used the experimental recording (upper trace) to constrain the cell model (middle trace) in response to the same current injection (lower trace). Sources for the experimental data: CA3 pyramidal cell [40], DG granule cell [41], OLM cell [42], and basket cell [43]. The parameter values for the model cells are listed in Table 1.

**Fig 2:**
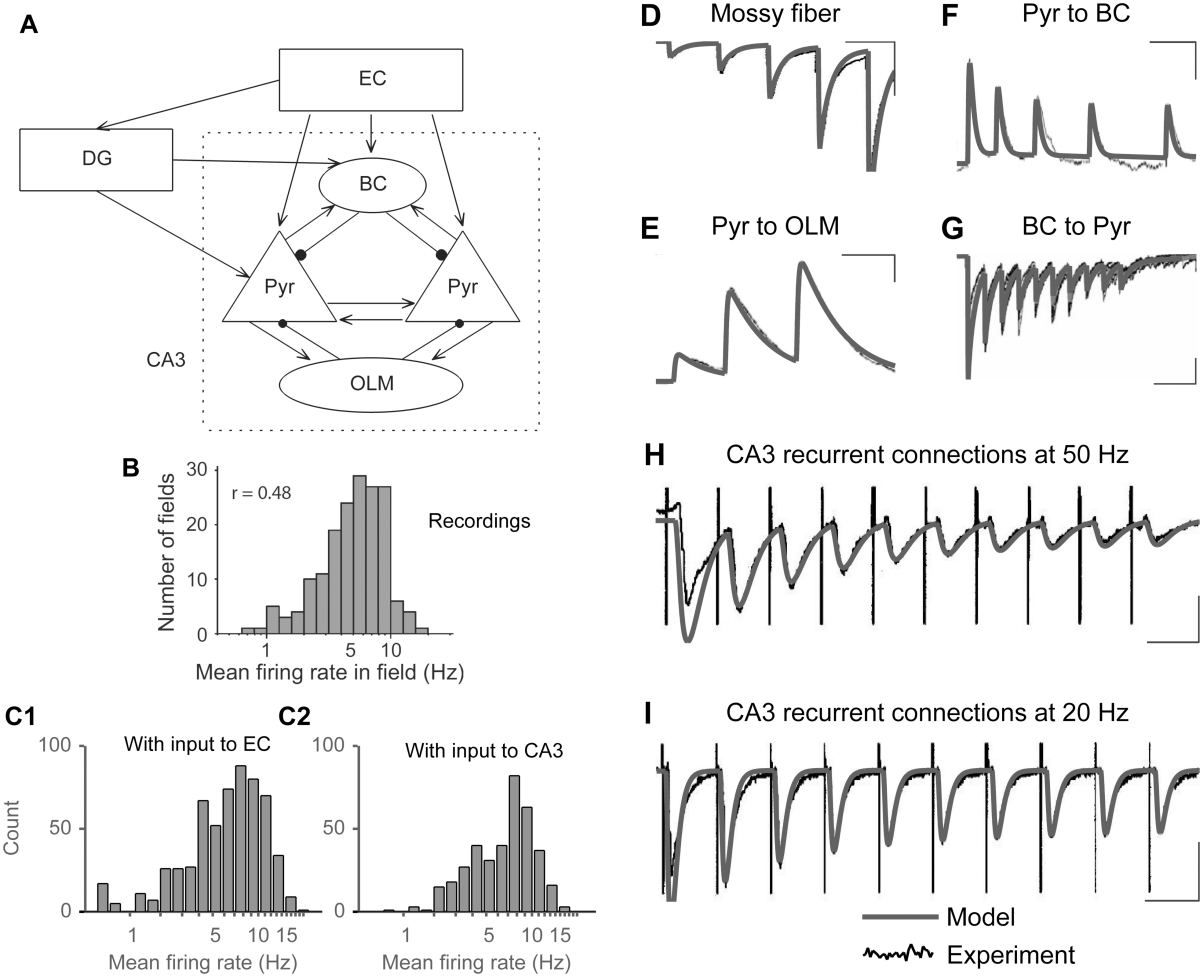
Network synaptic connections, titration of external input synapses, and the dynamics of short-term plasticity. A) Schematic showing the full model. Abbreviations: Pyr, pyramidal cells; OLM, oriens-lacunosum moleculare cells; BC, basket cells; EC, Entorhinal cortex; DG, dentate gyrus. B) *In vivo* distribution of CA3 place cells firing rates as the rat crossed their place field. Reproduced from [44]. C1) The distribution of CA3 pyramidal cells firing rates in the model case where random trains of synaptic inputs arrived at EC cells at a base rate of 15 Hz. C2) The distribution of CA3 pyramidal cells firing rates in the model case where random trains of synaptic inputs arrived at CA3 pyramidal cells at base rates drawn from a lognormal distribution with an average of 50 Hz and a standard deviation of 40 Hz. D-I: Synaptic model responses match those in experimental recordings. D) Mossy fiber synaptic facilitation [45]. (Scale bars: 50 ms, 100 pA). Parameter values used to reproduce data are listed in Hummos et al. [38]. E) CA3 Pyramidal cell to OLM interneuron [42]. (Scale bars: 20 ms, 1 mV). F) CA3 Pyramidal cell to BC interneuron [46]. (Scale bars: 30 ms, 0.5 mV). G) BC interneuron to CA3 pyramidal cell [47]. (Scale bars: 50 ms, 100 pA). H, I) Recurrent CA3 connections stimulated at 50 Hz, and 20 Hz, respectively [48]. Note that these connections displayed paired pulse facilitation, a phenomenon not included in our synapse model. Therefore, responses to the first stimulus in the train appear larger than in the recordings. (Scale bars: 20 ms, 0.5 mV in E; 50 ms, 0.5 mV in F).

We investigated theta generating mechanisms in the hippocampus using our published biophysical model that included networks for entorhinal cortex (EC), dentate gyrus (DG), and CA3 regions, and their interconnections [38]. The single cell models in the network were developed using the Izhikevich formulation [39] and matched to experimental recordings (Fig 1, see Methods for references and details). Cells were distributed in 3D space and connected following experimentally reported hippocampal spatial organization with lamellar connectivity between regions (see Methods). Basket cells (BCs) received input from EC, DG and CA3 pyramidal cells, while oriens-lacunosum moleculare (OLM) cells were reciprocally connected to pyramidal cells (Fig 2A, see Methods). Synaptic currents had values for rise and decay time constants obtained directly from published experiments (see Methods), and also exhibited short-term synaptic plasticity (Fig 2D–2I). The model was constrained further to perform pattern separation and completion, and was validated by its ability to match the effects of acetylcholine (ACh) in biasing the CA3 network towards pattern separation [details in 38].

Results reported below represent data averaged over 10 instantiations of the network with different random seeds for initial cell membrane potentials, synaptic connections, synaptic weights, and random external inputs. To examine the non-rhythmic component of medial septal and EC input, we used an input layer with 30 spiking neurons with no spike-frequency adaptation (referred to as ‘EC’). External input arrived as Poisson spikes to either this layer or directly to pyramidal cells depending on the experiment setup, at rates constrained to produce reported firing rates of hippocampal place cells during active locomotion [44; see Methods for details].

### Poisson spikes input generates theta oscillations in the model

**Fig 3:**
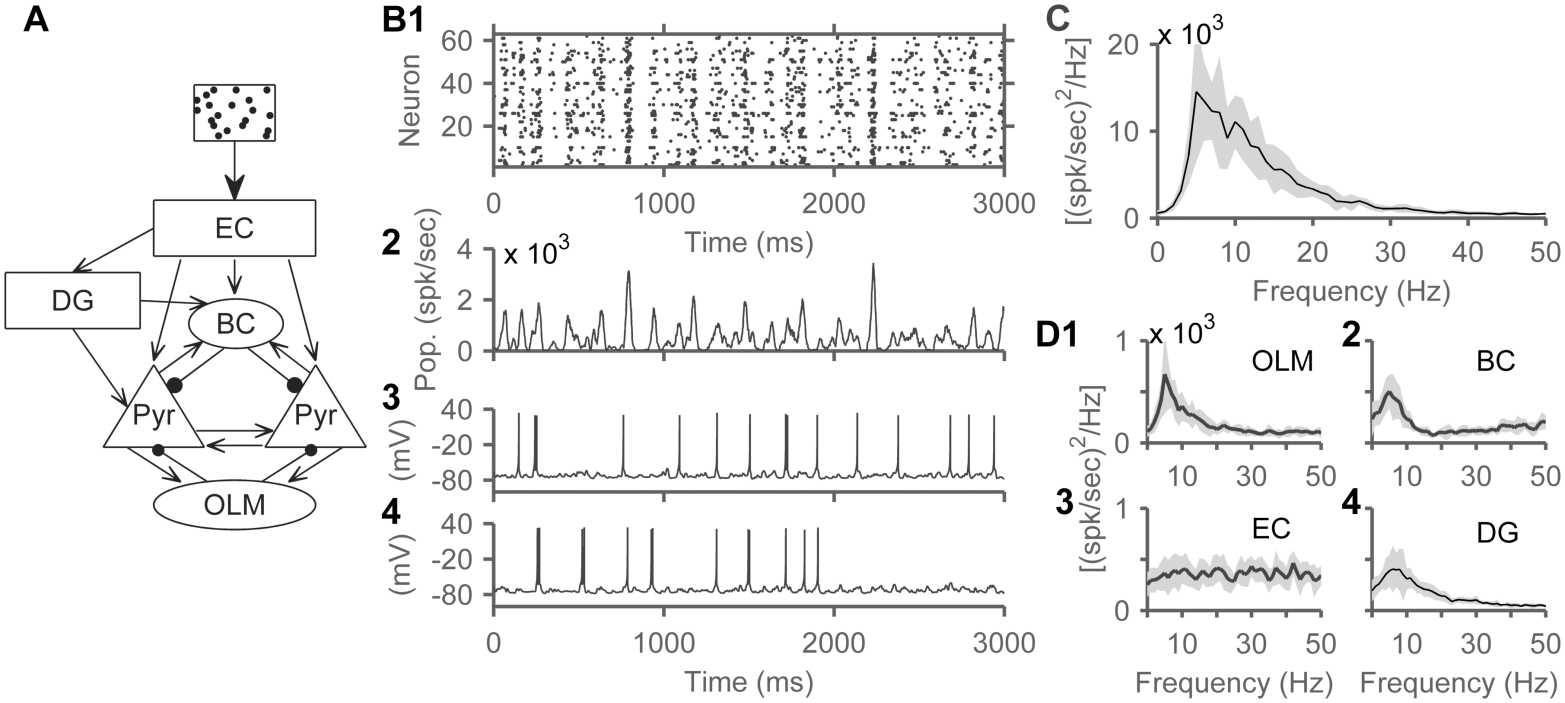
Model network displayed theta rhythmicity. External inputs arrived at EC cells in the form of Poisson spikes with a base rate of 15 Hz, chosen to reproduce place cells firing rates (see Methods). A) Network schematic showing the entire model. External input represented by box with random spike trains. B1) Spike raster plot from an example instantiation of the network. B2) P opulation firing rate calculated using a 20 ms sliding window and B3, 4) two membrane potential traces of neurons from the same network instantiation. C) The population power spectrum of CA3 pyramidal cells averaged from 10 network instantiations showing a peak at around 6 Hz. We sampled spike counts from the cell types considered, in 0.1 ms bins, and calculated the Fourier transform of the resulting vector, using a window length of 1024 ms. Shaded areas represent SD. D) Power spectra from four populations of cells in the model showed a peak around 6 Hz: (D1) OLM cells, (D2) BCs, (D3) EC, and (D4) DG.

The full model (Fig 3A) generated theta rhythmic activity, in response to external input in the form of Poisson spikes arriving at EC cells. Poisson inputs arrived at a rate of 15 Hz chosen to match experimentally recorded hippocampal firing rates (see Methods, Fig 2C1), but theta activity was robust over a range of input rates tested (5–100 Hz). Oscillatory activity was discernible in a spike raster plot of CA3 pyramidal cells from an example run, with multiple vertical groupings indicating synchronized firing (Fig 3B1). The population firing rate, calculated using a 20 ms sliding window (Fig 3B2), and the membrane voltage traces of two example neurons from the same run showed grossly rhythmic activity (Fig 3B3 and 3B4). The population power spectrum of CA3 pyramidal cells, averaged across 10 network instantiations, showed a consistent peak in the theta band (Fig 3C). Spiking activity of both inhibitory slow-spiking oriens-lacunosum moleculare (OLM) cells and fast-spiking basket cells (BC) also had a theta spectral peak (Fig 3D1 and 3D2), consistent with interneurons locking to theta rhythm in vivo [5]. The power spectrum of EC neurons spikes showed no distinct peaks confirming that the network received random Poisson-distributed inputs from EC (Fig 3D3). Finally, the DG area of the model, which contains granule cells reciprocally connected to the slow inhibitory HIPP interneurons, and basket cells, also generated its own theta rhythm (Fig 3D4).

Previous studies have suggested multiple potential theta generators in the hippocampus [1,49], so we examined which generators were engaged in our model by studying sub-networks with only a set of model components active during a specific run. Since rhythmic theta activity was also generated in DG (Fig 3D4), inputs from DG to CA3 were also disconnected in the studies reported below. Similarly, since the h-current is capable of producing theta resonance in any model sub-network [50–53], it was also removed, and the remaining cell currents were tuned again to match current injection data in Fig 1, without an h-current (cell parameters in Methods, as previously published [38]).

### Pyramidal cells display theta spiking oscillations

**Fig 4:**
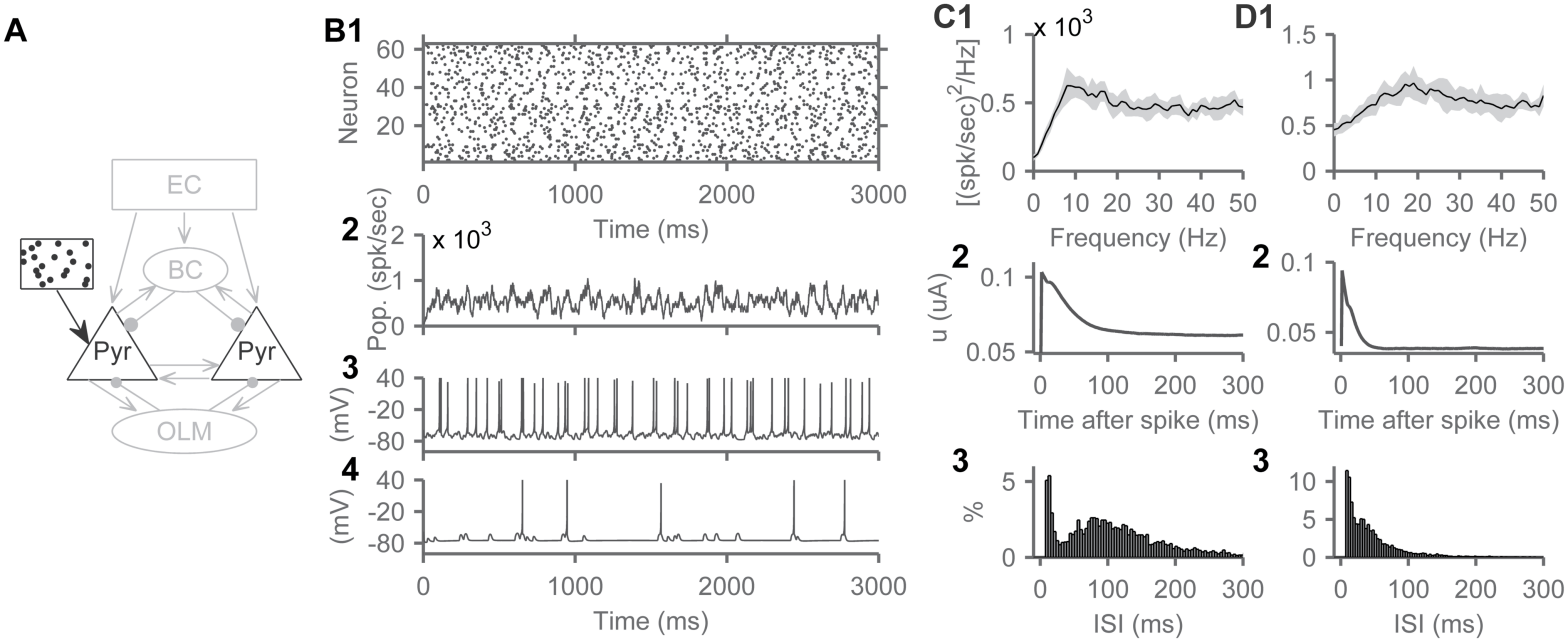
Disconnected pyramidal cells show theta spiking oscillations. A) Schematic of this experiment with isolated pyramidal cells each receiving a unique train of Poisson spikes with base rates derived from a lognormal distribution (mean 50 Hz SD 40 Hz, chosen to reproduce place cells firing rates). B1) Spike raster plot of pyramidal cells showing grossly random firing. B2) Population firing rate calculated using a 20 ms sliding window. B3, 4) Example membrane voltage traces from two neurons. C1) The power spectrum of the spiking activity of pyramidal cells as a population showing a peak at 8 Hz. C2) the spike-triggered average of the ‘u’ current of the Izhikevich model that captures the spike-frequency adaptation of the cells. C3) The inter-spike interval distribution of pyramidal cells. D) Same as in C, but with the time-constant of adaptation lowered from 100 ms to 25 ms, resulting in a power spectrum peak at 17 Hz, to demonstrate the relationship between the spiking oscillations, adaptation current, and the spiking dynamics of pyramidal cells.

The simplest case examined had isolated pyramidal CA3 cells (Fig 4A). The cells were completely disconnected, and each received a distinct train of Poisson spikes, at rates drawn from a lognormal distribution (to reproduce place cells firing rates, Fig 2C2). While their spiking activity appeared grossly random (Fig 4B), the power spectrum of the population, averaged over 10 instantiations of the model, peaked in the theta range (Fig 4C1). To investigate the underlying mechanisms, we considered the relationship between this power spectrum peak and the spike-frequency adaptation of pyramidal cells. The reader is reminded that this version of the model lacks an h-current, but focuses on modeling spike-frequency adaptation. In the Izhikevitch cell model, spike-frequency adaptation current is modeled by the second current ‘u’ (see Methods). A spike-triggered average of this current showed that it builds up after a spike or burst of spikes and decayed slowly going back to its baseline in about 90–100 ms (Fig 4C2). Hence, these cells had the highest probability of spiking again only after decay of the adaptation current, as confirmed by the inter-spike interval distribution peaking around 90 ms (Fig 4C3). This predominance of spikes occurring at theta intervals reflects a coherence resonance [54,55], which, in this context, can be defined as an oscillatory response, and a power spectrum peak (resonance), optimized by random perturbation in an adaptive cell. In other terms, the predominance of theta interval spiking could be interpreted as noise-induced “spiking oscillations”, as opposed to subthreshold oscillations. We will use the term “spiking oscillations” in this manuscript.

To further explore the relationship between spike-frequency adaptation and spiking oscillations, we lowered the time constant of adaptation from 100 ms to 25 ms (the parameter ‘a’ of the Izhikevich cell increased from 0.01 to 0.04, for this experiment, see Methods). We observed that the spiking oscillations shifted to higher frequencies around 17 Hz (Fig 4D1). The adaptation current decayed faster following a spike and the cells fired at shorter intervals (Fig 4D2 and 4D3). The adaptation time constant in our model cell (100 ms), chosen to match the current injection data from Brown and Randall [40, Fig 1], was comparable to the time-constant of adaptation recorded from hippocampal pyramidal cells (126 ms) [56]. Increasing the resting membrane potential of the cells had similar effects (not shown), due to the interaction of the potential with the spike-frequency adaptation current.

The reported power spectrum peak (Fig 4C1), which might be labeled as “spurious correlations” in digital signal processing, closely reflects basic properties of the cells that make up the network. The independent firing of neurons may not be a ‘mechanism’ of theta oscillations, due to absence of coordinated network activity. But such spiking activity can contribute to LFP once received by local cells, and should contribute to theta power measured from neuronal tissue. We next show how other network structures can exploit these spiking oscillations to generate robust theta activity.

### Divergent projections from EC produce theta oscillations

**Fig 5:**
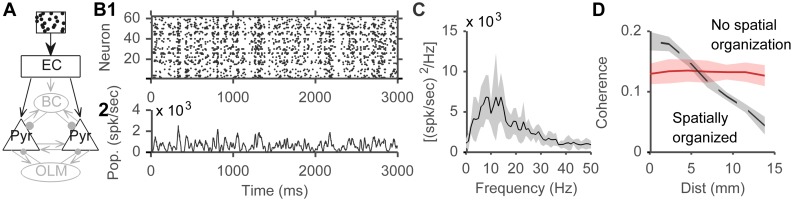
Divergent projections from EC to CA3 produced theta oscillations. A) Schematic of this experiment where the only active connections were the projections from EC to CA3 pyramidal cells. Model EC neurons lacked any rhythmicity and on average, each EC neuron projected to about 20% of CA3 pyramidal cells (see Methods). B1) Spike raster plot and (B2) population firing rate from one example network. C) The population power spectrum of CA3 pyramidal cells averaged over 10 network instantiations showed a wide based peak in the theta band centered around 9 Hz. D) Pairwise coherence between pyramidal cells is inversely related to the distance separating them (dashed black line), but this relationship is lost when EC neurons were connected to pyramidal cells randomly irrespective of the longitudinal distance (solid red line).

A novel finding from the model was that divergent projections from EC exploited the spiking oscillations cited in the previous section to produce theta activity, in a disconnected population of pyramidal cells. In this experiment, CA3 pyramidal cells received inputs from EC, but remained disconnected from each other (Fig 5A). All other cell types (OLM cells, BCs, and DG granule cells) remained inactivated, so pyramidal cells had no means of communicating with one another. EC cells were devoid of any rhythmicity (see Methods and Fig 3D3) and received Poisson spikes as external input, while CA3 pyramidal cells received input exclusively through the projections from EC. On average, each pair of CA3 pyramidal cells shared 19% of their projecting EC cells.

In this configuration, pyramidal cells spiking patterns appeared remarkably organized (Fig 5B), despite the absence of local connectivity in CA3. The power spectrum of the cell population confirmed rhythmic activity in the theta range (Fig 5C), with a power peak much higher than the power peak of independently firing pyramidal cells in Fig 4. The spatially divergent projection from EC to CA3 (see Methods) causes pyramidal cells in CA3 to have a degree of shared (correlated) input, and synchronizes the theta firing produced by their spiking oscillations. This mechanism has been reported in the generation of gamma oscillations in the piriform cortex [37].

Projections from individual EC cells to CA3 followed a Gaussian function of the longitudinal distance, creating neighborhoods in the longitudinal dimension [see Methods;, 57]. To examine its possible role, we removed the longitudinal organization by allowing EC cells to connect to any CA3 cell irrespective of their longitudinal location, while keeping the total number of connections constant. This randomization redistributes correlation across neuronal pairs, removing high input correlation between neighboring CA3 cells and the low input correlation for distant ones, and instead produces consistent correlation levels between all pairs of neurons at an average value. We observed the same power spectrum peak in the theta range (not shown), indicating that the specific longitudinal organization was not critical for the synchronized theta firing in CA3. Nonetheless, examining spiking coherence, as a measure of neuronal synchrony, showed that longitudinal organization created local areas of synchrony (Fig 5D).

### Recurrent connections and spike-frequency adaptation interact to generate theta oscillations

**Fig 6:**
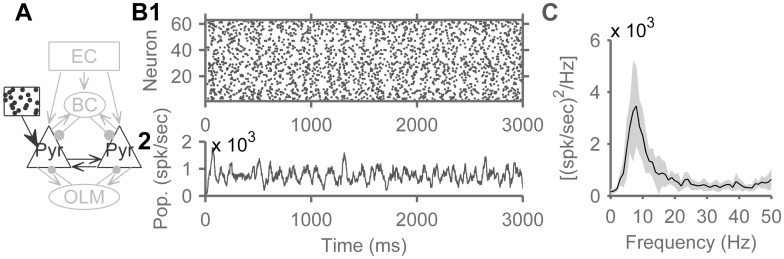
Recurrent connections produced theta oscillations. A) Schematic of the minimal network used for this experiment with only pyramidal cells and the synapses connecting them. B1) Spike raster plot and (B2) population firing rate from one example network. Note that in this sub-circuit, the oscillations are easier to identify visually in the firing rate plot. C) The population power spectrum of CA3 pyramidal cells averaged over 10 network instantiations shows a peak at 8 Hz.

We then investigated whether the excitatory connections between pyramidal cells in our model might synchronize their firing, as reported in previous computational studies [9,10,20,22,23,58]. For this experiment, the only active network components were CA3 pyramidal cells and the recurrent synapses connecting them (Fig 6A), and the h-current in pyramidal cells remained inactivated.

External inputs were applied directly to CA3 pyramidal cells instead of EC cells because the EC-CA3 pathway may have an independent role in the generation of theta rhythms, as discussed above. Power spectra averaged over 10 network instantiations showed a robust peak in the theta band (Fig 6B). This variation of our model closely resembles previous models [9,10,21,58,59], where spike-frequency adaptation interacts with the recurrent connections to produce theta oscillations.

### Pyramidal-interneuron sub-networks generate theta through two mechanisms

**Fig 7:**
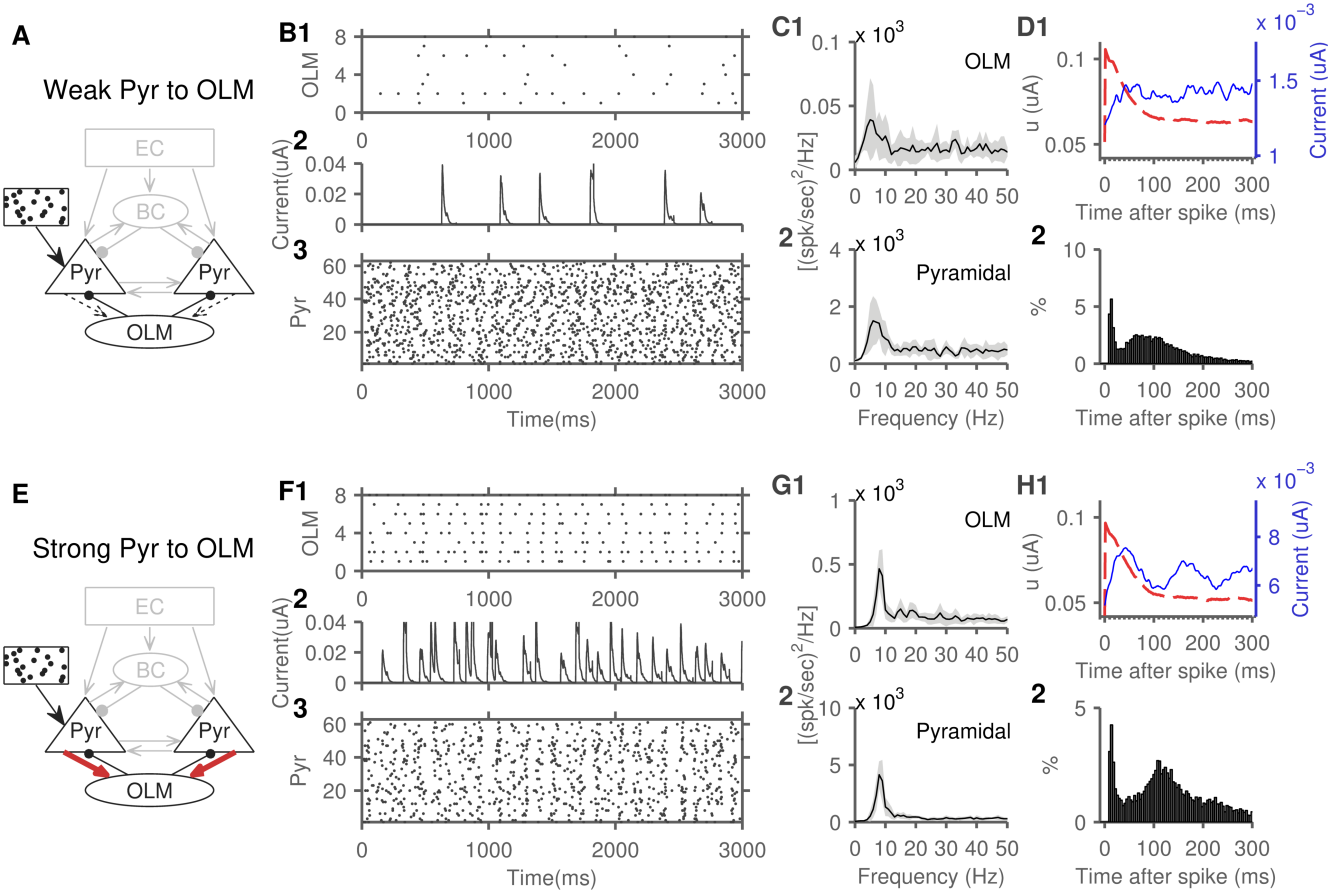
Pyramidal-OLM cells network generates theta through two mechanisms. A) Schematic showing the pyramidal-OLM cells network with weak pyramidal to OLM synapses (weight set to 2), used in panels B-D. B) Spike raster plot of OLM spikes (B1). The OLM inhibitory currents received by one example pyramidal cell (B2). Note that inhibitory currents occur at a frequency much lower than theta. Spike raster plot of pyramidal cells spikes (B3). C) The population power spectrum of OLM cells (C1), and pyramidal cells (C2). Spectrum averaged from 10 instantiations of the network, shaded areas represent SD. D1) The pyramidal cell spike-triggered average of the adaptation current (dashed red line) and received OLM inhibition (solid blue line). Note that OLM inhibition showed a poor association with pyramidal cells spikes. D2) the inter-spike interval distribution of pyramidal cells remains largely unchanged from freely spiking pyramidal cells (compare with Fig 4C3). E) Schematic showing the network with strong pyramidal to OLM synapses (weight set to 6), used in panels F-H. F1) Spike raster plot of OLM spikes. F2) The OLM inhibitory currents received by one example pyramidal cell. F3) Spike raster plot of pyramidal cells spikes. G) The population power spectrum of OLM cells (G1), and pyramidal cells (G2). H) The pyramidal cell spike-triggered average (H1) of the adaptation current (dashed red line) and received OLM inhibition (solid blue line). Note the close association between OLM inhibition and pyramidal cells spikes. Note that OLM inhibition changes the distribution of the pyramidal cells inter-spike intervals (H2), creating a peak around 110 ms. Power spectrum peaks: C1,2: 6 Hz. G1,2: 8 Hz.

We next examined the role of interneurons in rhythm generation. A sub-network of pyramidal cells reciprocally connected to a population of OLM cells generated robust theta oscillations (Fig 7), and varying the weight of the synapses from pyramidal to OLM cells illustrated two theta mechanisms in this sub-network. In the first case, with weak pyramidal to OLM cells synapses (weight set to 2, Fig 7A), OLM cells fired sparsely and sent inhibitory currents to pyramidal cells at a rate much lower than theta frequency (Fig 7B). However, theta oscillations emerged (Fig 7C) because of the pyramidal cells spiking oscillations, and the sporadic OLM inhibition acting as a common input [60]. In our model, any two pyramidal cells shared, on average, 32% of their OLM inputs. A spike-triggered average of OLM inhibitory currents showed minimal association with pyramidal cells spikes (Fig 7D1). Pyramidal cells continued to display the highest probability of firing at around 90 ms after a spike, when their adaptation current decayed to baseline (Fig 7D1 and 7D2). Similar “excitation-dominated” oscillations were shown in reduced models of this circuit [61].

In the second case, with strong pyramidal to OLM cells synapses (weight set to 6, Fig 7E), OLM cells fired near theta frequencies and generated theta-locked inhibitory currents in pyramidal cells (Fig 7F). OLM inhibition associated closely with pyramidal cells spikes, and shifted the pyramidal cells inter-spike interval peak to around 110 ms, to coincide with the trough of OLM inhibition (first trough at 110 ms and second around 220 ms).

**Fig 8:**
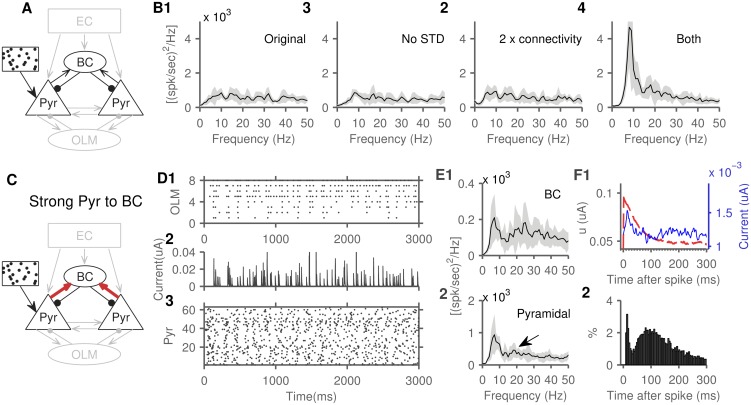
The role of BCs in theta rhythm generation. A) Schematic of this network with only pyramidal and basket cells active. B) In the original state, the sub-network failed to generate a significant spectral peak (B1, beyond the small theta peak expected from isolated pyramidal cells activity, see Fig 4). Oscillations remained absent after either removing short-term depression (B2) or doubling connection probability in both directions (B3). Both interventions were necessary to produce robust oscillatory activity (B4). C) Schematic of the network with strong pyramidal to basket cells synapses (weight set to 6), used in panels D-F. Note that short-term depression remained inactivated, and connectivity doubled for this experiment as well. D) Spike raster plot of BC spikes (D1). The BC inhibitory currents received by one example pyramidal cell (D2). The spike raster plot of pyramidal cells spikes (D3). E) The population power spectrum of BCs (E1), and pyramidal cells (E2), arrow pointing to a small emerging 18 Hz peak. F) The pyramidal cell spike-triggered average (F1) of the adaptation current (dashed red line) and received BC inhibition (solid blue line). Note the BC inhibitory current peaking sharply and then decaying back to average around 40 ms. F2) The inter-spike interval distribution of pyramidal cells. Power spectrum peaks: B1-3: 8 Hz. B4: 8 Hz, 18 Hz. E1: 6 Hz, 23 Hz. E2: 6 Hz, 18 Hz.

In contrast, the BC-pyramidal cells sub-network is viewed as a generator of gamma oscillations [for review, see 62]. In a simulation that had only BCs and pyramidal cells and their interconnections (Fig 8A), our first unexpected finding was that this sub-network did not generate any rhythmic activity (Fig 8B1). We discovered that two characteristics of the connections between BC and pyramidal cells prevented an oscillatory coupling: short-term depression [46,47], and the lower connection probability compared to that of OLM-pyramidal connections [63,64]. We observed that removal of short-term depression and increase of connection probability (doubling) were both required for oscillatory activity (Fig 8B2–8B4). The second unexpected finding, however, was that this oscillatory activity was in the theta range (Fig 8B4).

As demonstrated in the OLM-pyramidal sub-network, weak pyramidal to BC drive can potentially produce sporadic BC inhibition with the sole effect of synchronizing the theta spiking oscillations of pyramidal cells, independent of the specific properties of BC inhibition. But, even with a higher pyramidal to BC connection weight (set to 6), we continued to observe theta oscillations (Fig 8C–8F). To explore the lack of gamma activity, we examined the spike-triggered average of both the adaptation current and the BC inhibitory current in pyramidal cells. To generate gamma activity, pyramidal cells are expected to spike again as BC inhibition decays, however, the ISI distribution showed that cells are most likely to spike again after their own adaptation current decays (Fig 8F). Thus, pyramidal cells adaptation dominated the dynamics of the sub-network due its slower dynamics, thereby generating oscillations that depend primarily on the dynamics of pyramidal cells rather than the dynamics of inhibitory synapses. Similar pyramidal cells driven oscillations has been seen in a model of the neocortex [65]. Of note, there was an emerging peak at around 17 Hz in the power spectrum of pyramidal cells (Fig 8E2, indicated with arrow). An upcoming study of this model investigates the role of background synaptic input in enhancing and shifting this peak to gamma frequencies, similar to the findings of Economo and White [65].

### Theta generators have different relative contributions across cholinergic states

Next, we examined the effects of inactivating individual theta generators, across different cholinergic states. We started with the full model with external random inputs arriving at EC (DG area and the h-current remained inactive). We then inactivated each theta generator individually and observed changes in the relative theta power (ratio of power in the 4–12 Hz range to the entire spectrum from 0 to 250 Hz). To inactivate the spiking oscillations of pyramidal cells we removed spike-frequency adaptation by lowering the adaptation time constant to 10 ms (cell parameter ‘a’ set to 0.1, see Methods) so that it recovered promptly after each spike. The contributions of the recurrent connections, OLM cells, and BCs were removed by inactivating the corresponding synapses. To remove the effects of the projections from EC, we replaced them with direct random inputs to CA3 pyramidal cells that achieved the same firing rate (input rates drawn from a lognormal distribution with mean (± SD) of 50 Hz ± 40 in baseline ACh, 20 Hz ± 20 in low ACh, and 60 Hz ± 50 in high ACh).

**Fig 9:**
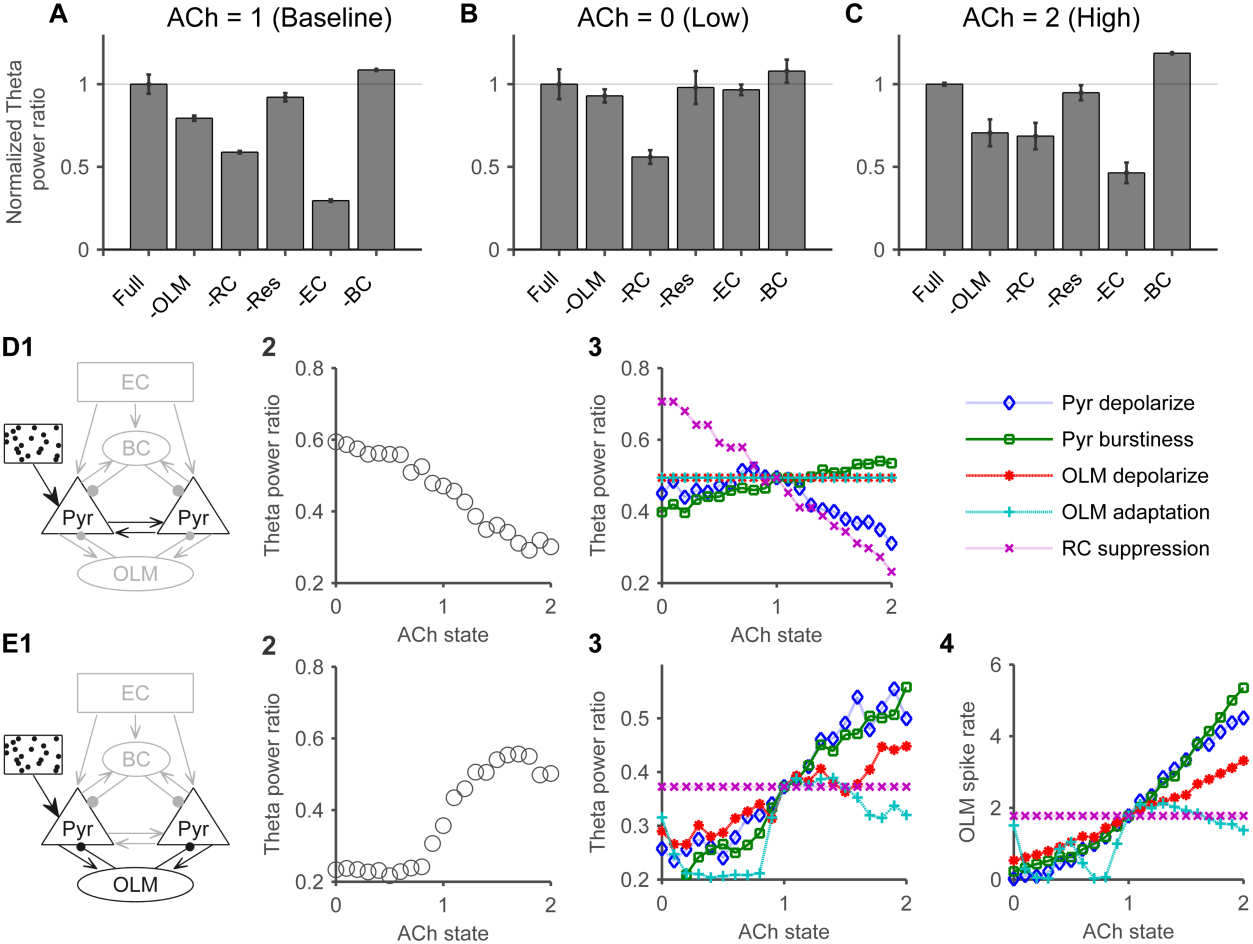
Relative contributions of individual theta generators across cholinergic states. A) Starting with the full EC and CA3 circuits we inactivated each individual generator and observed the changes in relative theta power (power in the 4 to 12 Hz range divided by the entire spectrum 0 to 250 Hz). To normalize, all values were divided by the relative theta power in the full model. Error bars indicate SD. B) The effects of inactivating individual generators in the low cholinergic state, and C) high cholinergic state. D) In the recurrent connections sub-network (D1), relative theta power decreased with higher cholinergic states (D2). A breakdown of cholinergic effects (D3) revealed that this was mainly mediated by the cholinergic suppression of the recurrent connection transmission (RC suppression) in addition to the cholinergic depolarization of pyramidal cells (Pyr depolarize). ACh in increments of 0.1. E) Schematic of the OLM-pyramidal cells network (E1). Relative theta increased with cholinergic state (E2) and a breakdown of cholinergic effects (E3) shows that cholinergic suppression of spike-frequency adaptation in OLM cells (OLM adaptation), and OLM cells depolarization (OLM depolarize) enhanced theta power in the network. In addition, the cholinergic pyramidal cells depolarization (Pyr depolarize) and enhanced burstiness (Pyr burstiness, lower c and d parameters of Izhikevitch model) enhanced theta in this network with a steeper slope. These effects were associated with enhanced OLM cells firing rates (E4).

In the baseline cholinergic state, removing EC projections had the most profound effect on relative theta power. This large drop in relative theta power suggests a prominent role for the non-rhythmic component of external input from medial septum or EC in the generation of hippocampal theta *in vivo* (Fig 9A), considering that EC activity was dominated by non-rhythmic input (see Methods). The relative contribution of EC projections was followed by that of the recurrent connections, and then OLM cells (Fig 9A). Interestingly, inactivating the spiking oscillations of CA3 pyramidal cells had minimal effects on relative theta (Fig 9A), presumably due to compensation by the other generators. Removal of BC inhibition slightly raised relative theta (Fig 9A), due to lowered feedforward inhibition from EC (average pyramidal cell firing rate increased from 7 Hz to 9 Hz).

We repeated the analysis for the low and high cholinergic state networks. Acetylcholine (ACh) state affected the cells and synapses of the network and took values from 0 (lowest) to 2 (highest, see Methods). The low cholinergic state increased the impact of removing the recurrent connections and decreased the impact of removing OLM cells (Fig 9B). The high cholinergic state produced the opposite effects (Fig 9C). To focus on CA3 dynamics, DG was not included in the simulations in Fig 9. A separate simulation examined the effects of adding DG input and showed a significant increase in CA3 relative theta power only in high cholinergic states (relative theta increase in low cholinergic state: 0.01, p < 0.5, med: 0.02, p < 0.5, high: 0.15, p < 0.05).

These inactivation results are suggestive but not conclusive, due to compensatory changes in the network, so we examined the effects of ACh on these specific theta generators in isolation (Fig 9D and 9E). The sub-network of recurrently connected pyramidal cells generated the highest theta power in the low cholinergic states (Fig 9D2), whereas the OLM-pyramidal cells sub-network had its theta peak in the high cholinergic states (Fig 9E2). A similar analysis of the EC induced oscillations showed no significant relationship between cholinergic modulation and theta power.

Cholinergic stimulation in the model had several effects on the neurons and synapses of the network (see Methods). We ran simulations by isolating individual cholinergic effects and allowing only one effect to be expressed in each run. Cholinergic suppression of the recurrent connections, and the cholinergic depolarization of pyramidal cells impaired theta generation in the recurrently connected pyramidal cells sub-network (Fig 9D3). While it was initially counterintuitive as to why depolarizing pyramidal cells would impair theta generation, we noted that raising the resting potential in our model also controlled the dynamics of the spike-frequency adaptation current (see Eq 2), and adaptation is required for theta generation in a recurrently connected network [9,10,21,58]. Cholinergic effects on OLM cells (depolarization and reduced spike-frequency adaptation) enhanced theta in the OLM-pyramidal cell sub-network, but cholinergic effects on pyramidal cells (depolarization, and enhanced burstiness) had an even steeper effect (Fig 9E3), presumably due to the short-term facilitation at the pyramidal to OLM synapses. These effects were mediated by enhanced OLM cells firing rates (Fig 9E4).

In summary, the inactivation of individual theta generators had a variable impact on theta power as a factor of the state of cholinergic neuromodulation. The recurrent connections played a major role in the low cholinergic state while OLM-pyramidal cells sub-network played a more substantial role in the high cholinergic state.

## Discussion

A biophysical model of the hippocampus provided an integrative understanding of theta generation and, for the first time, examined five cooperative generators of theta activity. Furthermore, it helped reveal variable engagement of the theta generators in different neuromodulatory states. The model was developed by matching biological data including single cell behavior, synaptic dynamics, connectivity patterns, and short-term synaptic plasticity. In a previous study, the model parameters were constrained to replicate pattern completion and separation behavior in the hippocampus, and subsequently validated by testing the model’s ability to reproduce the effects of ACh in biasing the CA3 network towards pattern separation [38]. The model was used here as previously published with no changes in parameters (available at the public site ModelDB; see Methods). The present study sheds light on several underlying mechanisms involved in theta generation, as discussed below.

### Multiple solutions, one rhythm

Earlier reviews speculated several intrinsic theta generators in the hippocampus [1,49]. Our results are consistent with this account, and further provide a tool to examine the relative contributions of each generator and to shed light on how the generators cooperate and compensate for one another. The exceptional robustness of hippocampal theta generation suggested that the rhythm can be considered as an intrinsic property of the network. Consequently, any experimental manipulation or brain state that generates sufficient excitation in the hippocampus may produce theta oscillations, non-specifically. This is consistent with experimental reports of oscillatory activity being generated by simply enhancing neuronal excitability by distributed electrical excitation [66], or by raising extracellular potassium concentration [67].

The abundance of theta generators suggests that designing studies to establish the necessity or the sufficiency of one factor to theta rhythms might be difficult in experiments. For example, in a recent study, optogentic silencing of OLM cells in vivo did not diminish theta activity [8]. In light of our results, this lack of effect on theta rhythm is expected, and is not evidence against a role for OLM cells in theta rhythm generation. In fact, within the integrative framework presented here, disruption of theta after a particular generator is inactivated would warrant a closer inspection, since causes for the absence might include extreme neuromodulatory states, or diminished excitation below a minimum threshold.

The robustness of theta generation might seem to contradict the presence of other non-theta states. However, it is important to note that we constrained the firing rates distribution to match that of place cells during locomotion, a state characterized by prominent theta activity. In addition, we expect neuromodulation to strongly influence the repertoire of oscillatory states in the hippocampus.

### Relative contributions of different generators

We quantified the relative contributions of theta generators by inactivating each individually and assessing the relative drop in theta power (Fig 9A–9C), generating several relevant insights. For example, we found that generators can have a variety of interactions such as compensating for inactivated generators, as exemplified by the network’s ability to compensate for the removal of pyramidal cells resonance (Fig 9A and 9C). Importantly, we also showed that removal of the divergent projections from EC to CA3 produced the highest drop in theta power (Fig 9A and 9C), suggesting that despite the local circuit theta generators, external input amplifies the rhythm substantially. The fact that our model EC neurons lacked rhythmic properties (Fig 3C3) highlights a role for solely the strength of the external input, i.e., without a frequency-modulated component. This observation is of relevance when considering the substantial drop in hippocampal theta observed after inactivating external input from EC [31] or the medial septum [29]; external input strongly synchronizes hippocampal rhythms. Our results provide a more nuanced interpretation of these experimental findings [29,31], where receiving external signals, even if randomly distributed, synchronizes hippocampal rhythms. This expands our understanding of the role of these external inputs beyond the interpretation that hippocampal theta is dependent on receiving theta-modulated external input from either the medial septum or EC. Computational modeling in this case allowed us to delineate two effects of external input that might be difficult to dissociate experimentally and suggested that both random input as well as frequency-modulated input have independent contributions to hippocampal theta.

Significantly, this methodology sheds some light on the mechanisms most crucial to theta generation across neuromodulatory states. We previously showed that the low and the high cholinergic states engendered run away excitation via distinct pathways, and also differed in the mechanisms that contained such aberrant excitation [38]. The present study revealed parallel differences and extended this perspective to suggest that the two circuits also differ fundamentally in how they generate rhythmic activity, consistent with the suggested role of neuromodulation in profoundly reconfiguring neuronal circuits [68–70]. This conceptual delineation might be more tied to the underlying mechanisms compared with the categorization of theta into atropine-resistant and atropine-sensitive forms [71]. Furthermore, using this framework, more detailed conductances-based models might reveal how specific neuronal conductances are involved in theta generation across cholinergic states, since these specific currents are the targets of cholinergic modulation either directly (for e.g. m-current) or indirectly (e.g. h-current, through changes in resting potential).

### Intrinsic theta generation with no local connectivity

An interesting observation from the model was that a power spectrum peak could be detected in the activity of a population of disconnected and independently firing neurons [Fig 4;, 72]. Indeed, the power spectrum of summed independent signals is proportional to the autocorrelation function of individual neurons [73, page 184]. A novel finding from the model was to demonstrate that these correlations, though sometimes disregarded as an artifact, had the highest impact on theta power, when entrained by shared extrinsic input from EC (Fig 9A).

Coordinated activity through input correlations has been observed experimentally in the olfactory cortex [37], but has not been studied in the hippocampus. The spatial divergence of the projections between layers causes cells in the target layer to share many of the same inputs. So, although the EC inputs themselves are not correlated per se, their spatial projection to CA3 pyramidal cells results in the latter having correlated inputs. Since CA3 pyramidal cells had spiking oscillations at theta, this sharing of inputs caused coherent firing at a similar phase, generating coherent theta oscillations [74], despite the absence of any local connectivity. Any resonant process in CA3, such as the h-current in pyramidal or OLM cells or the OLM-pyramidal cell subnetwork [3,6,14,15], can be organized by the shared external input, to generate robust rhythmic activity. While the observed oscillations had a wide-based spectral peak, the architecture of the hippocampus with multiple layers providing divergent projections from one to the next [57] can amplify this effect at each layer, resulting in a robust method for generating rhythms.

### The role of pyramidal-interneuron sub-networks in oscillatory activity

Computational models of theta generation emphasized the role of OLM cells, and in particular their h-current, in rhythm generation [3,6,14,15], but recent evidence has suggested a more modest h-current in OLM cells [7]. To reconcile these findings with computational models, we simulated the OLM-pyramidal cells sub-network with inactivated h-current (to take the recent findings to their extreme) but still observed theta generated through two other mechanisms. First, their slow inhibition can form a theta-generating feedback loop with pyramidal cells (Fig 7A–7D) [14,15,20,58]. Second, OLM cells can generate theta by merely synchronizing the activity of the theta resonant pyramidal cells (Fig 7A–7D). Simulating this sub-network with an active h-current enhanced theta power as shown in previous models [3,15,75].

We propose considering these two mechanisms and the h-current as three distinct mechanisms in the OLM-pyramidal sub-network, probably operating simultaneously to maintain theta rhythmic activity. Another mechanism that is relevant to purely inhibitory networks is theta generated by a population of recurrently connected OLM cells sharing inhibitory input [14], but our model lacked the necessary OLM to OLM connections to test the presence of this mechanism.

In the model pyramidal-BCs sub-network, two modifications were necessary to obtain oscillatory activity (Fig 8A and 8B). The connection probability between the two cell populations had to be increased above our initial estimates, similar to previous modeling studies [15]. While this might be an issue of modeling at a lower scale compared to biology, it might suggest a lower participation of BCs in oscillatory activity, compared to OLM cells, since they send and receive relatively fewer connections to and from pyramidal cells [63,64]. In addition, we show that increased connectivity was not sufficient by itself to produce oscillatory activity, but suppression of short-term synaptic depression, a cholinergic effect [76], was also required (Fig 8B3 and 8B4).

While fast inhibition is associated with the generation of gamma oscillations [61,62,77], a prediction of the model is that the BC-pyramidal cell sub-network can also, indeed, support the generation of ~8 Hz rhythmic activity in states with active spike-frequency adaption in pyramidal cells (Fig 8). This behavior might be modulated in states where spike-frequency adaptation is suppressed [for e.g., by neuromodulation 78–80]. We also observed an emerging peak around 17 Hz which might be a precursor to gamma-range oscillations when the network is subjected to sufficient background activity [65].

### Putting it all together

Rhythmic oscillations in multiple frequency bands are associated with the functioning of most nervous systems, and are a topic of intense research. Underlying such oscillatory activity are complex interactions at multiple levels including individual neurons, local circuits, and neuronal systems. The multitude of generators and interactions amongst areas makes the system particularly difficult to investigate experimentally. We believe that our study contributes to the rapidly growing literature in neural oscillations. Specifically, we suggest how activity in any region could be studied as being comprised of three components, an intrinsic ability of the region to generate oscillations, a random non-rhythmic extrinsic component that coordinates oscillations generated intrinsically in the region, or/and a frequency-modulated rhythmic extrinsic component that specifically entrains neurons in the region to a particular frequency. This idea holds, in general, to both other forms of oscillations and other brain regions. Moreover, it could be relevant to other biological phenomena involving oscillations.

We suggested how the role of the intrinsic biophysical mechanisms in such complex oscillatory systems can be studied using a biologically realistic model. We devised a simple and experimentally practical approach through serial inactivation of individual rhythm generators across neuromodulatory states. This simplified approach yielded significant insights showing that different neuromodulatory states may engage different theta generators. Additionally, we showed that external input can have a prominent contribution to theta power in the hippocampus, but also that the hippocampus is not solely dependent on this external input being theta-modulated.

The model makes five experimentally testable predictions. First, stimulation of EC in an in vitro preparation of the hippocampus is sufficient for generating oscillatory activity in CA3 even in states with diminished local synaptic transmission in CA3. Second, OLM cells can generate theta activity via at least two mechanisms: synchronizing pyramidal cells with a common inhibitory signal, and pacing theta activity through slow inhibitory feedback loops. Third, BCs reciprocally connected to pyramidal cells are capable of generating theta activity by virtue of spike frequency adaptation in pyramidal cells. Fourth, since the spiking oscillations demonstrated in model pyramidal cells relies on their ubiquitous spike-frequency adaptation, neuromodulators that affect this adaptation should significantly modify resonance characteristics in biological cells. Fifth, while impairment of any single theta generator might not disrupt rhythmic activity, conditions can be set up through neuromodulation to emphasize one generator over the others. For example, inactivation of OLM cells would have a larger effect in high cholinergic states, while inactivation of recurrent connections would affect theta prominently in low cholinergic states. Modern tools, such as optogentics, with the ability to control specific pathways, would facilitate experimental testing of these predictions.

## Methods

### Single cell models

The model cells in CA3 were pyramidal cells and two of the most abundant interneuron types, BCs and OLM cells [81]. The two types of interneurons are on extreme ends of many cellular attributes such as spiking patterns, inhibition dynamics and post-synaptic target compartments, and so their inclusion captures a wide range of interneuronal dynamics. The model cells in DG were granule cells, BC, and hilar perforant path-associated (HIPP) cells.

Single cell models were developed using the Izhikevich formulation [39,82]. The equations for a model neuron were as follows:

$$\frac{dv}{dt}=-k\left(v-v_{t}\right)\left(v-v_{r}\right)-\;u+I$$ (1)

$$\frac{du}{dt}=-a\left(b\left(v-v_{r}\right)-u\right)\;$$ (2)

 if v>vpeak then v=c and u=u+d  (3)

where *v* is the membrane potential of the cell, *u* is a recovery variable, *v*_*t*_ is the ‘instantaneous threshold’ beyond which the cell will fire an action potential, *v*_*r*_ is the resting membrane potential, *I* is the current injection, *k* is a constant used to adjust the input resistance and rheobase, *vpeak* is the threshold above which a spike is deemed to have occurred and the membrane potential is reset, and *a*, *b*, *c*, and *d* are parameters used to tune the behavior of the system to model the neuro-computational properties of the desired cell. While the NEURON environment is typically used for Hodgkin-Huxley cell models, we developed a biophysical cell model in NEURON and implemented the Izhikevich formulation by adding a current modeled by the two equations.

This formulation provides a reduced-order model that preserves many of the neuro-computational properties of more detailed biological models. We provide an overview below of how model neurons were developed to match salient features in electrophysiological recordings (Fig 1), with parameters used listed in Table 1. For CA3 pyramidal cells, the resting membrane potential was set to -75 mV, spike threshold to -53 mV, and peak action potential voltage to 29 mV [40]. The remaining cell model parameters were tuned to match responses to both long and brief current injections (Fig 1) [40]. Similarly, in developing the DG granule cells model, resting membrane potential, threshold, and peak action potential were set using data from Staley et al., [41] and the model was then tuned to match current injection responses (Fig 1) [41]. Passive properties for the OLM model were estimated from Ali and Thompson [42], and the behavior of the cells was matched to current injection responses from the same study. In particular, we matched the spike frequency adaptation, the prominent slow after-hyperpolarization potential (AHP), sag response, and rebound spikes (Fig 1). For the BC model, membrane properties, current injection responses (Fig 1), and finally current vs. firing rate relationship were matched to data reported in Buhl et al., [43]. Due to the striking similarity of OLM and HIPP cells [83], we used the same model for both cell types. EC cells are known to display theta rhythmicity [31]. So, to examine the non-rhythmic component of EC input and its interaction with the intrinsic generators of theta in the hippocampus, we excluded oscillatory dynamics in EC cells by using generic non-adapting spiking cells [82].

**Table 1:** Model cells parameter values.

**Parameters** **Cell types**	**C (pF)**	**k**	**a (1/ms)**	**b**	**c (mV)**	**d**	**v**_**r (mV)**_	**v**_**t (mV)**_	**v**_**peak (mV)**_
**CA3 pyr**	24	1.5	0.01	2	-63 ^a^	60	-75	-58	29
**GC**	24	1	0.015	3	-62	3	-73	-53	32
**O-LM**	80	1.5	0.003	10	-65 ^b^	30	-68	-53	30 ^b^
**BC**	16	1.5	0.9	2	-80	400	-65	-50	28

^a^ for the burst response to brief current pulse, c was -53

^b^ for OLM interneurons, c and v_peak_ were dependent on the recovery variable u, where c was incremented by (10 * u) and v_peak_ was decremented by (30 * u) to produce the stereotypical shape of OLM firing.

Despite the significant heterogeneity of neurophysiological values reported across studies, our model neurons preserve the most salient cellular features in relative terms. For example, OLM interneurons fire at a slower rate than basket cells [81], and CA3 pyramidal cells burst more than granule cells of the dentate gyrus [84]. Such relative attributes of the cells are well-preserved in our model, irrespective of the particular set of neurophysiological values chosen. Other experimental data considered in developing the single cell models can be found in Hummos et al. [38]. Initial membrane potential values were drawn from a normal distribution with a mean equal to the resting membrane potential and a standard deviation of 10 mV. The h-current in pyramidal and OLM cells is known to have a role in theta generation [for review, see 2], so we added an additional slow current to our pyramidal and OLM cells tuned to match the subthreshold oscillations that the dynamics h-current produces [16]. The additional current equation took the form:

$$\frac{dh}{dt}=-a_{h}\left(b_{h}\left(v-v_{r}\right)-h\right)$$ (4)

where *h* is the h-current value, and *a*_*h*_, *b*_*h*_ are parameters used to tune the behavior of the cell and took the values of 0.04 ms^-1^ and 10 for pyramidal cells and 0.03 ms^-1^ and 3.5 for OLM cells. A reset parameters *d*_*h*_ was added to the value of *h* each time the cell spiked and took a value of 1 for both pyramidal and OLM cells. These values were chosen to match the subthreshold resonance reported in literature for these two cell types [16].

### Network structure and connectivity

The rat hippocampus contains approximately 1.6 million cells [85]. For computational efficiency and to maintain minimum model complexity, the numbers were scaled down while maintaining reported ratios [38], as in our previous models [86–89]. The model DG region had 384 granule cells, 32 BCs, and 32 HIPP interneurons, while the model CA3 region contained 63 pyramidal cells, 8 BCs, and 8 OLM cells [85,90–92]. The model EC region had 30 regular spiking cells.

The entorhinal cortex provides inputs to the hippocampus through the perforant pathway that projects to the entire hippocampal formation. The standard view describes a unidirectional connectivity with a direct path from EC to CA3 and an indirect path through DG (Fig 2A and 2B) [57,93]. The perforant path projections follow a lamellar organization across the longitudinal axis of the hippocampus, as follows: Lateral and posterior parts of the EC are connected to the dorsal parts of CA3 and DG, while the more medial and anterior parts of EC project to the ventral parts of CA3 and DG [57]. This lamellar organization transitions gradually from one extreme to the other on the longitudinal axis of the hippocampus, and a single neuron in EC can project to about 25% of the longitudinal length of CA3 [57]. Projections from DG to CA3 also follow a similar longitudinal organization; however, these projections target a more limited longitudinal extent [57].

Model cells were distributed uniformly in 3D space separated into the three regions, EC, DG, and CA3, with dimensions that approximate the respective dimensions of the rat hippocampus [38]. Projections from EC to both pyramidal cells and BCs in DG and CA3 followed a lamellar pattern where neurons were most likely to connect to neurons in of their longitudinal neighborhood with a decreasing probability towards the periphery. This spatial connectivity was modeled using a Gaussian connection probability function that depended on the longitudinal distance between the two connected cells. The Gaussian function had a peak probability of 0.4 and a standard deviation of 3 mm for the perforant path projections to both pyramidal cells and BCs in CA3. Perforant path projections to DG had similar values (see [38]).

Similarly, the mossy fiber projections from DG to CA3 followed the same lamellar pattern but with a more limited longitudinal extent by setting the standard deviation of the Gaussian probability function to 2 mm. In addition, to preserve the sparseness of the mossy fiber connections from DG to CA3 [57], each DG granule cell was limited to contacting two CA3 pyramidal neurons. Projections from DG granule cells to CA3 BCs are more diffuse and out-number projections to CA3 pyramidal neurons by a ratio of 10:1 [94]. Accordingly, DG projections to BC followed a Gaussian distribution with a peak probability of 0.2 and standard deviation of 3 mm. Recurrent CA3 connections reveal relatively more diffuse spatial organization [95,96], and were therefore distributed homogenously with a fixed probability of 0.3.

The dendritic projecting OLM cells are thought to be involved in feedback inhibitory loops [97] and while they have a more limited axonal arborization [98] they make many more synapses compared to BCs [63]. In contrast, BCs have a more diffuse axonal arborization with the highest connection probability to pyramidal cells in their immediate neighborhood and a decreasing connection probability towards the periphery of their axonal arbors [63]. Similarly, BCs project to neighboring OLM cells [99]. As before, we used a Gaussian function to approximate these spatial probabilities. We also assumed that BC projections to both pyramidal cells and to OLM cells shared the same spatial domain. In the reverse direction, OLMs receive reciprocal connections from the same pyramidal cells they projected to, in line with their function as local feedback cells [97]. On the other hand, granule cells in DG and pyramidal cells in CA3 projected homogenously to BCs with a fixed probability of 0.15, consistent with the lack of specific topography reported at these projections [100].

The network was constructed by generating connections randomly between cells while maintaining the connection probabilities and spatial patterns of connectivity described above. The spatial connectivity patterns and parameter values are summarized in Table 2 (also see [38]).

**Table 2:** Summary of synaptic properties used in the CA3 network model.

	EC input	Recurrent	Pyr to OLM	Pyr to BC	OLM to pyr	BC to pyr
Spatial connectivity	Diffuse [57]	Homogenous [96]	Reciprocal [97]	Homogenous [100]	Dense, compact [102]	Light, diffuse [98]
*Synaptic Delay (ms)*	5 [104,105]	2 [48]	0.9 [103]	0.9 [103]	0.8 [103]	0.8 [103]
AMPA or GABA_A_ rise/decay time constants (ms)	1.7/10.9 [102] ^a^	1.1/5 [48]	0.27/0.57 [103]	0.27/0.57 [103]	2.8/20.8 [99] ^a^	0.21/3.3 [99] ^a^
NMDA or GABA_B_ rise/decay time constants (ms)	25/300 [102]	25/300 [102]	25/150 [102,106]	25/150 [102,106]	11.1/125 [101]	11.1/125 [101]
Weight	2	0.4	3	3	3	3
Short-term synaptic plasticity	None	Depressing [48]	Facilitating [46]	Depressing [46]	None	Depressing [47]
bACh	-0.5[107]	-0.85 [107]	None	None	None	-0.5 [108]

bACh: a unit-less value determining the direction and magnitude of ACh effects on synapses (see Methods)

^a^ We calculated the rise time constant from the reported 20–80% rise time or 10–90% rise time, see Hummos et al. [38].

### Synaptic currents

Synaptic currents were modeled using the kinetic model described in Destexhe et al. [101]. AMPA, NMDA, GABA_A_, and GABA_B_ currents were modeled and their dynamics such as rise and decay time constants and delays were matched to available literature [38]. In particular, CA3 pyramidal cell AMPA currents were fastest for the mossy fiber inputs from DG and slowest for perforant path inputs from EC, while recurrent CA3 inputs from other pyramidal cells had intermediate values [48,102], as summarized in Table 2. Additionally, inhibitory currents from OLM had slower dynamics compared to those from BC (Table 2) [99,103].

Synaptic weights were assigned in accordance with literature where available. The mossy fiber synapses were adjusted so that a train of spikes arriving at the synapse could cause a CA3 pyramidal cell to fire while a single spike could not [109]. Recurrent CA3 connections were assigned a low initial weight, as an approximation of data showing that the transmission of action potentials had a probability of 4% at those synapses [110]. Synapses from CA3 pyramidal cells to interneurons were set at a higher level to reflect the fact that action potential transmission occurs at a ~60% success rate [111,112]. Connections between granule cells and DG interneurons were adjusted to achieve sparse DG firing [113]. Synapses had initial weights chosen from a uniform random distribution of values between 50% and 100% of the assigned weight value. All associated equations and parameter values are described in Hummos et al. [38].

### Activity-dependent plasticity

For this study, long-term plasticity was excluded from the synapses. Model synapses, however, exhibited short-term synaptic plasticity that used the formulation proposed by Varela et al. [114]. We modeled the pronounced short-term facilitation at mossy fibers [45] and the frequency-dependent synaptic depression reported at the recurrent CA3 connections [Fig 3D–3I;, 48]. We extrapolated from data in CA1 where projections from pyramidal cells display short-term facilitation at synapses contacting OLM cells [42], and short-term depression at those contacting BC cells [46]. In the other direction, inhibitory currents from OLM cells to pyramidal cells show no short-term facilitation or depression [97], while inhibitory currents from BCs to pyramidal cells show depression [47]. Model traces are compared with available experimental recordings in Fig 2. Additional details and equations related to the implementation of short-term synaptic plasticity can be found in Hummos et al [38].

### Acetylcholine effects

The hippocampus receives widespread volume transmission of cholinergic inputs from the septum-diagonal band complex [115]. To implement the effects of ACh on model neurons and synapses, we used a variable ‘ACh’ to represent the ACh state. The variable ACh had values of 0 (low), 1 (baseline), and 2 (high).

Cholinergic stimulation has differential effects on synaptic transmission of different pathways in the hippocampus [116]. Synaptic transmission through the perforant pathway projections to CA3 is suppressed by 50%, compared to a suppression by 85% at the recurrent connections in CA3 [107,117]. On the other hand, the mossy fibers transmission is enhanced by 49% [118]. To model ACh effects on synapses, AMPA synaptic currents were scaled by the value of ACh. A parameter bACh determined the direction and magnitude of ACh effects on a particular synapse. Values of bACh for different synapses were set according to experimental results as summarized in Table 2 (also see Hummos et al. [38]).

In addition to the synapse specific effects, cholinergic stimulation enhanced cellular excitability and depolarized the resting membrane potential of principal cells, eliminated AHP, decreased spike frequency adaptation and induced rhythmic burst activity [78,79]. Furthermore, effects on interneurons were subtype-dependent [119,120]. Muscarinic stimulation of OLM interneurons depolarized the resting membrane potential, and lowered both spike frequency adaptation and AHP [121]. In contrast, PV-BCs respond to muscarinic receptor activation with a limited depolarization in resting membrane potential [122,123]. Effects of ACh on neurons were modeled by linearly scaling the neuronal model parameters by the ACh state as detailed in Hummos et al. (see Fig S4 in [38]). Considering the slow dynamics of ACh effects [onset time constant approximated between 1 and 2 s;, 124], ACh state was set to a given value at the beginning of each experiment and had no dynamics.

### Inputs and data analysis

For the full model and sub-circuit cases considered, either EC cells or CA3 pyramidal cells (identified in the figures) received external input as trains of Poisson-distributed spikes, triggering an influx of AMPA and NMDA currents into the cell. We studied two model cases: one with external input arriving at EC, and the other with input arriving directly at CA3 pyramidal cells. The two types of inputs differed in the weight of the associated input synapses, and the base rate of the Poisson spike trains arriving at these synapses. Input to EC arrived at synapses with a 100% spike transmission rate to ensure that EC firing pattern was dictated by the Poisson input, whereas input to CA3 pyramidal cells had a lower weight value with parameters matching the EC to CA3 synapses (Table 2).

To determine the base rates of the Poisson processes generating these input trains, we considered place cells in CA3. Place cells respond to certain areas in the environment and their firing rates approximate a lognormal distribution [44] with an average of ~7 Hz [125]. In our model case where external inputs arrived at EC, each EC cell received a unique train of Poisson input spikes at a base rate of 15 Hz, which produced firing rates in CA3 pyramidal cells with a lognormal distribution and an average of 7 Hz (Fig 2C1). In the model case where external inputs arrived directly to CA3 pyramidal cells, the input rates to different cells had to be drawn from a lognormal distribution (average: 50 Hz, standard deviation: 40 Hz), to produce firing rates with a lognormal distribution (Fig 2C2) that matched experimental data.

For spectral analysis, we summed the spikes of all cells of each type in a region (e.g., CA3 pyramidal cells) in 0.1 ms bins and computed the fast-Fourier transform of the resulting vector, using the Matlab function psautospk.m [126], with a moving window of size 1024 ms, and overlap of 512 ms. Spike data was used in spectral calculations as a proxy for LFP as used in network models [e.g., 127–129], with the assumption that these spikes are received by a downstream local neuron and translated into membrane currents that generate an LFP signal.

The coherence measure used was implemented as described by Wang and Buzsaki [72]. Briefly, the coherence measure reflects synchrony between the spike trains of two neurons (two spikes are synchronous if occurring within 5 ms of each other). The population coherence is the average coherence across all neuron pairs to give a measure between zero (minimum synchrony) and one (maximum network synchrony). Oscillatory activity tends to synchronize the network making coherence a good measure of oscillations.

The model was developed using the NEURON software package [130] and run on a PC with an Intel i7-core processor with an integration time-step of 0.1 ms (key results were also verified with a time-step of 0.01 ms). The code is available as part of our previous publication via the public database ModelDB at Yale University. The recorded spike times were then analyzed using MATLAB (Mathworks, Inc.). All simulations ran for 5 seconds except for the experiment in Fig 4, where single neuron spike data was analyzed over a 30 second period.
